# The success of opening concurrent chronic total occlusion lesion to improve cardiac function trial in patients with multi-vessel disease (SOS-moral): Study protocol of a prospective multicenter study

**DOI:** 10.1097/MD.0000000000020349

**Published:** 2020-05-22

**Authors:** Jinfan Tian, Huijuan Zuo, Lijun Zhang, Mingduo Zhang, Dongfeng Zhang, Min Zhang, Yuan Zhou, Yi He, Hongzhi Mi, Xueyao Yang, Rongchong Huang, Xiantao Song

**Affiliations:** a Department of Cardiology; b Department of Community Health Research; c Department of Radiology, Beijing Anzhen Hospital; d Department of Radiology, Beijing Friendship Hospital; e Department of Nuclear Medicine, Beijing Anzhen Hospital, Capital Medical University, Beijing; f Department of Cardiology, The First Affiliated Hospital of Dalian Medical University, Dalian; g The Department of Cardiology, Beijing Friendship Hospital, Capital Medical University, Beijing, China.

**Keywords:** cardiac function, cardiovascular magnetic resonance, chronic total coronary artery occlusion, coronary artery disease, multi-vessel coronary artery disease, percutaneous coronary intervention

## Abstract

**Aims:**

The purpose of the present trial is to determine whether opening co-existing chronic total occlusions (CTOs) using percutaneous coronary interventions (PCIs) improves cardiac function in patients with multi-vessel disease (MVD). Patients with MVD are defined as having at least one additional major vessel exhibiting no less than 75% stenosis combined with the presence of a CTO artery.

**Methods and results:**

Patients will be prospectively recruited who meet the following criteria:

(1) angiographically diagnosed with MVD concurrent with CTO;
(2) previously diagnosed with myocardial infarction, or suffered myocardial necrosis of the CTO-territory determined using cardiovascular magnetic resonance (CMR).

Patients presenting with no necrosis of myocardial tissue in the territory of the CTO will be excluded. Recruited patients will be randomized into 2 groups: those undergoing PCI of only the non-CTO artery (non-CTO PCI group), and those undergoing PCI of the non-CTO artery concurrently with the CTO artery (CTO-PCI group). The primary outcome will be the change in cardiac function evaluated via CMR at a 12-month of follow-up appointment, which will be compared to a baseline measurement. Secondary outcomes include occurrence of major cardiac events, CMR-assessed myocardial viability in the CTO-supplied territory, and quality of life assessed by Seattle angina questionnaire, Patient Health Questionnaire 9 and Generalized Anxiety Disorder Scale-7 after 12-month follow-up.

**Conclusion:**

The SOS-moral trial will provide data necessary to determine whether to open concurrent CTOs among MVD patients with CMR-detected necrotic myocardial tissue.

## 1 Introduction

A chronic total occlusion (CTO) is defined as a coronary artery occlusion for a period ≥3 months, which can be detected by angiogram.^[1]^ The rate of CTO detected by angiography among patients diagnosed with coronary artery disease is approximately 10% to 30%.^[1,2]^ A recent study that recruited 14,441 multi-vessel disease (MVD) patients revealed that those with a concurrent CTO lesion have higher rates of mortality than those not presenting with concurrent CTO (Hazard Risk: 1.29, *P* < .01). The difference was most significant in young patients and those with acute ST-segment elevated myocardial infarction (STEMI).^[3]^ Another study found that ischemic cardiac dysfunction was associated with increased mortality among patients with CTO lesions within the first 1 to 2 years of identification of the disease.^[4]^ Percutaneous coronary intervention (PCI) for CTO is performed to relieve symptoms, improve cardiac function, decrease the need for subsequent coronary artery bypass graft (CABG), and improve quality of life (QOL).^[5]^

Previous studies revealed that for both CTO of a single coronary artery and MVD with concurrent CTO, even with well-developed collateral circulation, successful CTO-PCI is associated with reduced long-term cardiac mortality and reduced need for CABG, which is an advantage that can persist 2 to 5 years following PCI.^[6–12]^ However, another study could not demonstrate benefits of CTO-PCI when assessing the occurrence of major adverse cardiac events (MACE); among patients with CTO of a single coronary artery, PCI did not reduce cardiac mortality and MACE when compared to optimal medical therapy (OMT).^[13]^ The prospective, open-labeled, randomized DECISION-CTO trial revealed that OMT as an initial strategy was non-inferior to PCI with regard to the composite endpoint of death, MI, stroke, or any revascularization within a 3-year follow-up period.^[14]^ A multicenter, open-labeled, randomized, controlled EuroCTO trial showed that PCI for CTO resulted in a significant improvement of health status compared with OMT alone, rather than resulting in the reduction of MACE.^[15]^

The presence of a CTO and not MVD alone has been reported to be independently associated with long-term mortality and impaired left ventricular eject fraction (LVEF).^[16]^ Hence, the effect of CTO-PCI on cardiac function merits investigation. An early study that recruited patients with at least 1 CTO and a LVEF of ≤ 40% found that CTO-PCI decreased left ventricular end-systolic volume and increased LVEF.^[17]^ Another study, with a small sample size, showed that successful CTO-PCI improved regional wall motion.^[18]^ The EXPLORE trial showed that routine CTO-PCI did not result in an overall benefit with respect to LVEF and lower left ventricular end-diastolic volume (LVEDV) within 4 months, when compared with a non-CTO PCI strategy in an unselected cohort of patients with STEMI and concurrent CTO.^[19]^ An extended examination of the EXPLORE trial also did not reveal beneficial effects associated with routine early CTO-PCI on LVEF within a 1-year period.^[20]^

Myocardial viability affects CTO-PCI outcomes. With respect to myocardial viability, the myocardium in the CTO territory can be divided into groups as follows: complete survival, partial survival, and non-viable myocardium. We predict that conflicting conclusions drawn by previous studies can be partially attributed to the failure to consider the status of myocardial tissue or the burden of ischemia.

At present, US and European guidelines provide Class IIa or IIb recommendations for PCI for the treatment of CTO. The recommendation comes despite the fact that the rate of successful CTO-PCI is more than 90% at experienced centers and can be increased by training of operators working within clinical practices, especially those in small centers. These facilities include a large portion of patients presenting with CTO combined with MVD who receive treatment involving exclusively non-CTO PCI or incomplete revascularization. The purpose of the present trial is to compare the effect of CTO-PCI combined with non-CTO PCI versus non-CTO PCI with respect to cardiac function, myocardial viability, and MACE among patients with MVD and necrotic myocardial tissue. Cardiovascular magnetic resonance (CMR) will be used to assess the viability of the myocardium, which facilitates identification of patients who will benefit from complete revascularization procedures.

## 2 Methods

### 2.1 Study design and randomization

This SOS-moral study is a randomized controlled trial with a multi-center design. The protocol was approved by the ethics committee of Beijing Anzhen Hospital (approval no. 2017059X) and has been registered at ClinicalTrials.gov (identifier: NCT 03372785).

**Figure 1:**
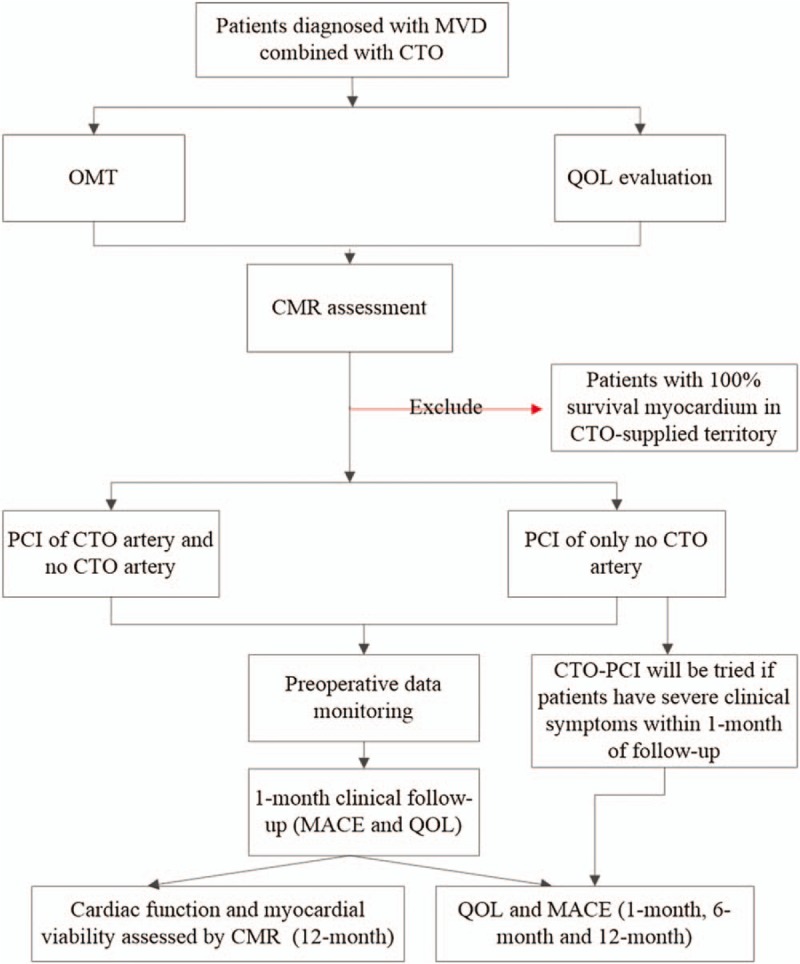
Flow chart of the study design. CTO = chronic total occlusion; MVD = multi-vessel disease, PCI = percutaneous coronary intervention; MACEs = major adverse cardiac events; CMR = cardiovascular magnetic resonance; QOL = quality of life; OMT = optimal medical therapy.

Patients meeting the inclusion criteria will be categorized into two groups at a ratio of 1:1 according to a randomized digital table used to randomly assign patients to the CTO-PCI group (PCI of both the non-CTO artery and a concurrent CTO artery), or the non-CTO PCI group (PCI of only the non-CTO artery). The randomized digital form and interactive voice response mode will be applied to facilitate the randomization procedure. The randomization sequence will be produced and managed by an independent staff member. Before intervention, myocardial viability and cardiac function of patients will be evaluated using CMR. Cardiac function will be assessed using transthoracic echocardiography and CMR. The Seattle angina questionnaire (SAQ), Patient Health Questionnaire 9 and Generalized Anxiety Disorder Scale-7 will be used to evaluate patient QOL. Cardiac function, QQL, and MACE will be observed within 12 months of follow-up. A flow chart outlining the study design is depicted in Figure 1.

### 2.2 Treatment groups

Patients in the CTO-PCI group receive PCI treatment involving both the CTO artery and the non-CTO artery. Patients of the non-CTO PCI group will only have PCI performed on the non-CTO artery. To ensure safety of all patients, they will be monitored, and those with persisting clinical symptoms occurring within one month of OMT will undergo PCI of CTO artery.

### 2.3 Patients and enrollment

We will enroll patients hospitalized for stable or unstable angina pectoris in Beijing Anzhen Hospital, The First Affiliated Hospital of Dalian Medical University and Beijing Friendship Hospital from April of 2018 to December of 2021 meeting the selection criteria. All subjects/guardians enrolled in the trial should clearly understand the research requirements and intervention procedures, and each will be required to sign a written informed consent agreement before participating in any type of examination or intervention. All participating research centers should obtain all informed consent forms from patients before the initiation of intervention. Personal information will be kept confidential before, during, and after the trial.

#### 2.3.1 Inclusion criteria

To meet inclusion criteria, patients should: be 18 to 80 years old; be diagnosed with single CTO concurrent with MVD detected using coronary angiography (at least one other major vessel should have exhibited no less than 75% stenosis); previously have been diagnosed with myocardial infarction, or suffer from myocardial necrosis in the territory of CTO artery determined by CMR; present with an LVEF above 35% determined using transthoracic echocardiography; present with CTO located in an epicardial coronary artery with a reference diameter of ≥ 2.5 mm; and comply with all the evaluations and follow-up protocols.

#### 2.3.2 Exclusion criteria

Patients will be excluded if they have suffered from acute myocardial infarction within the previous 3 months; a lesion located in the left main artery (stenosis ≥50%); rheumatic valvular disease; severe arrhythmia; a history of revascularization within the non-CTO artery; multiple vessels with CTO (more than one CTO artery); lesions unsuitable for PCI; severely abnormal hematopoietic systems, such as platelet counts <100 × 10^9^/L or > 700 × 10^9^/L and white blood cell counts < 3 × 10^9^/L; with active bleeding or bleeding tendencies (active ulcers, short-term ischemic stroke, history of hemorrhagic stroke, intracranial space occupying lesions, recent craniocerebral trauma, and other bleeding or bleeding tendency); severe coexisting conditions, including severe renal function dysfunction [Glomerular filtration rate less than 60 ml/min • 1.73 m^2^), severe hepatic dysfunction [glutamic-pyruvic transaminase (ALT) or glutamic-oxal acetic transaminase (ALT) elevated more than three times that of the upper limit of the normal reference], severe heart failure (NYHA classification III–IV), acute infectious diseases and immune disorders; tumors; surgery within 3 months; a life expectancy less than 12 months; pregnancy or planning to become pregnant; history of allergy or adverse reactions to aspirin, clopidogrel, ticagrelor, stains, contrast material, anticoagulant, or stents. Patients will also be excluded if they cannot tolerate dual antiplatelet treatment; are unable to communicate due to cognitive impairment, auditory, or visual impairment; are participating in another trial for medication or an apparatus and in which the main endpoint has not been reached, or plan to participate in a clinical trial within 12 months of the intervention.

### 2.4 Intervention

#### 2.4.1 PCI procedure

Experienced operators have been designated to ensure that the success rate of CTO-PCI is above 80%. Procedural success is defined as resulting in residual stenosis of <10% with thrombolysis in myocardial infarction grade 3 flow, in the absence of dissection and thrombosis. The use of a DES and specialized devices will be left to the discretion of the operator.

#### 2.4.2 Medication

Patients included in both CTO-PCI group and non-CTO PCI group will be administered aspirin (300 mg) and one P2Y12 receptor antagonist (300–600 mg clopidogrel or 180 mg ticagrelor) before PCI unless they have been treated with these antiplatelet agents previously. Calcium antagonists, nitrates, sodium nitroprusside, and GP IIb/IIIa receptor antagonists will be prepared and administered in any case in which no-reflow occurs in the PCI procedure. After PCI, all patients will be routinely administered one P2Y12 receptor antagonist (clopidogrel 75 mg/d or ticagrelor 90 mg twice daily), aspirin (100 mg/d) and statin once daily for 12 months. Other medications such as beta-blockers, calcium antagonists, and nitrates will be administered according to the clinical status of each patient, considering factors such as heart rate, blood pressure, and cardiac function.

### 2.5 Evaluation

#### 2.5.1 CMR scanning

CMR will be performed using a Siemens 3.0T whole-body MR scanner (MAGNETOM Verio, A Tim System; Siemens Healthineers, Erlangen, Germany). A 32-element matrix coil will be utilized for data collection, and electrocardiographic gating will be utilized to acquire all sequences. First, the morphologic data from the heart will be acquired with the short-axis plane (from the base to the apex) and long-axis plane of the left ventricle (LV). Next, 0.05 mmol/kg Magnevist contrast agent will be injected intravenously with a high-pressure syringe at 5 mL/s. The first-pass perfusion image (8-mm sections with no intersection gap) will be acquired when magnevist is injected. LGE imaging (8-mm sections with no intersection gap) will be acquired 10 minutes after the intravenous administration of gadolinium (Magnevist, Bayer Healthcare, 0.1 mmol/kg) using a 2-dimensional, phase-sensitive, inversion-recovery, breath-hold sequence.

CMR will be utilized to assess myocardial infarct size, left ventricular mass, LVEF, LVEDV, left ventricular end-systolic volume (LVESV), cardiac output, stroke volume, segmental wall thickening, and myocardial viability in the territory supplied by the artery with CTO.

#### 2.5.2 Transthoracic echocardiography

Transthoracic echocardiography (Philips ie33 GE vivid 7) will be utilized to determine left ventricular end diastolic diameter, left ventricular end systolic diameter, LVEF, and segmental wall thickening.

### 2.6 Experimental endpoints and procedural follow-up

Patients will be followed-up via outpatient consultation 1, 6, and 12 months following PCI. A follow-up appointment 12-months post-PCI will be conducted at the hospital. Basic characteristics including gender, BMI, waist circumference, comorbidities, medication history, blood pressure, and heart rhythm both at baseline and 1-year post-PCI will be documented by an independent physician. Blood tests assessing routine blood counts, liver and kidney function, blood lipid profiles, fasting blood glucose, glycosylated hemoglobin, hsCRP, and BNP will be recorded at baseline (before angiography) and at 1-year post-PCI. CK, CK-MB, and cTNI levels will be collected at baseline, one day after intervention, one week after intervention, and at 1-year post-PCI.

#### 2.6.1 Primary endpoint

The primary endpoint of this SOS-moral study will be difference in cardiac function observed between baseline and 1-year post-intervention, which will be determined by CMR and transthoracic echocardiography.

#### 2.6.2 Secondary endpoints

Secondary endpoints involve the occurrence of MACEs, defined as all-cause mortality, cardiac death, a first or recurrent, non-fatal, acute myocardial infarction, target lesion revascularization (PCI or CABG), stroke, heart failure and cardiac rehospitalization within 1, 6, and 12 months post-PCI. Other secondary endpoint will include changes in myocardial viability in the territory supplied by the CTO artery between the baseline and 12 months post-PCI, to be determined by CMR. In addition, QOL changes at 1, 6, and 12-month follow-up appointments will be assessed by comparing with baseline values using the SAQ, patient health questionnaire 9 and Generalized Anxiety Disorder Scale -7. Finally, the volume of contrast used during the procedure and the total cost of medical care covering OMT and the equipment for coronary artery disease therapy from the day of enrollment (first hospitalization) to the last follow-up (rehospitalization) will be evaluated. Perioperative myocardial infarction will be evaluated by assessing CK, CK-MB, and cTNI levels;

#### 2.6.3 Safety endpoints

The safety of the perioperative period, including acute coronary artery occlusion, acute vascular perforation, acute stent thrombosis, acute myocardial infarction, and cardiac death of the two groups assessed, will be compared.

### 2.7 Sample size calculation

PASS 15.0 will be utilized to calculate sample size. In the study, 226 individuals are predicted to be recruited in the trial. This sample size was calculated using the optimal efficiency test. The main purpose of this trial is to investigate the effect successfully opening concurrent CTO lesions on cardiac function in patients with MVD. LVEF has been taken into consideration for the calculation of sample size. Previous studies revealed a 6.5% mean difference between the two experimental groups and a 9% standard deviation with respect to LVEF. The optimal boundary value for LVEF was set to 4%.^[21]^ Two-hundred sixteen cases recruited to the study will allow for the use of a 1-tailed test with a significance level (α) set to 0.025, and power (1-β) of 0.90. Ultimately, it is estimated that 240 patients will be randomized, and a 10% loss is expected to occur throughout the 1-year follow-up period.

### 2.8 Statistical analysis

In the full analysis set, all randomized patients will be kept in the treatment group to which they were originally randomized. Moreover, patients without protocol deviation will be included in the per protocol set. Patients of the per protocol set will be included in baseline information analysis rather than in primary analysis if the CTO-PCI fails. Patients will not be included in the primary analysis if the MACEs occur.

Continuous variables normally distributed will be presented as means ± standard deviation, and student *t*-test will be used to make comparisons between two independent samples. Median and 4-quintile intervals will be used to express variables that are not normally distributed, and the Mann–Whitney *U*-test will be used to compare differences. Categorical variables will be presented as frequencies and percentages, and the differences between groups will be assessed using a chi-square test. Survival analysis will be performed using a Kaplan–Meier curve and Cox regression analysis. A 2-sided *P*-value < .05 will be considered statistically significant.

### 2.9 Data collection, monitoring, and quality evaluation

Inclusion and exclusion criteria will be enforced strictly to minimize the potential for the inclusion of confounders. An experienced cardiovascular interventionist (CTO-PCI >50 cases/yr) has been designated to ensure that the success rate of CTO-PCI is above 80%. The number PCI performed within each of the centers participating in this study is least 3,000 cases. An expert committee has been established to ensure interventional safety and prevent study-associated complications. At least two senior experts in PCI (PCI >3000 cases per year) will assist to discern MACEs throughout the follow-up period. Two experienced specialists will acquire CMR images and collect data using specific equipment and software. Two independent investigators will be responsible for the creation of protocols and supervising follow up, and they will communicate with the enrolled participants throughout follow-up. The case report form will be completed by independent investigators. All data obtained will be documented in the database by two independent investigators according to guidelines governing data management. A special inspector will be responsible for tracking, monitoring and reviewing the research process, and it will not be possible to alter data at will. The database will be inactive after cross validation, followed by statistical analysis. The staff that perform analysis will be blinded to the intervention.

## 3 Discussion

This trial aims to investigate the effect of CTO-PCI combined with non-CTO PCI on cardiac function in patients with MVD. Patients with MVD in a EuroCTO trial received non-CTO PCI before randomization; participants in the DECISION-CTO trial were not limited to those with MVD; the EXPLORE study recruited patients with STEMI. Consequently, a number of studies have investigated the clinical effects of successful CTO-PCI;^[6,10]^ however, there remains a lack of evidence from a randomized control trial, which can be sued to conclusively determine whether the success of opening concurrent CTO lesions improves cardiac function among patients presenting with MVD but not STEMI.

Early studies reported that successful recanalization CTO can both improve LVEF and ventricular wall movement assessed by CMR, and can improve QOL.^[22–25]^ An early meta-analysis found that successful recanalization of a CTO resulted in an overall improvement of 4.44% absolute LVEF points, reduced remodeling, and improved survival (OR: 0.52).^[26]^ However, the EXPLORE trial showed that in acute STEMI patients with concurrent CTO, CTO-PCI did not increase global LVEF or decrease LVEDV assessed by CMR 4-month post-PCI compared to patients receiving treatment in the non-CTO artery. Interestingly, a subgroup analysis of the EXPLORE trial showed that CTO-PCI in patients with a concurrent CTO in the LAD was associated with significantly higher LVEF levels after 4-month post-PCI compared with non-CTO PCI, suggesting that CTO-PCI may potentially benefit high-risk patients.^[19]^ According to Chung,^[27]^ the benefit of CTO-PCI is controversial with respect to cardiac function and survival among the MVD patients with concurrent CTO. This may partly be because myocardial viability affects the alteration of cardiac function produced by CTO-PCI. An in-depth analysis in the EXPLORE CMR study revealed that CTO-PCI was associated with the greater recovery of regional systolic function relative to baseline values 4-month post PCI in dysfunctional myocardial tissue supplied via CTO. The benefit was most evident in dysfunctional but viable segments (transmural extent of infarction <50%).^[28]^ The investigators opined that the benefit was probably attributable to the recovery of stunned myocardial tissue affected by the acute interruption of blood supply, as well as by the prevention of arrhythmia. Taken together, the data suggest that varying levels of myocardial viability in patients included in previous studies may be associated with reported variations in cardiac functioning observed after successful CTO-PCI treatments. Therefore, myocardial viability should be considered.

Previously, 128 patients were observed using CMR, and among the myocardial regions that corresponded to 149 CTO lesions, only 11.5% presented as transmural myocardial infarction. In fact, 58.6% of the myocardial segments showed no delayed enhancement.^[29]^ Differences in myocardial viability may contribute to the variable prognosis of CTO-PCI. A previous study including 32 CTO patients presenting with significant inducible perfusion deficits found that recanalization was associated with decreased ischemic burden, improved QOL, and reversed remodeling.^[23]^ The effect of CTO-PCI on cardiac function among patients with necrotic myocardial tissue in the territory supplied by the CTO combined with MVD requires further investigation. The present SOS-moral study excludes those patients with complete myocardial survival in the CTO artery territory. This distinction will facilitate the investigation of the effects of actively opening concurrent CTO lesion in MVD patients with necrotic myocardial tissue identified via CMR in the territory supplied by the CTO, aiming to provide reliable evidence for use in clinical practice.

CMR is an appropriate approach for the evaluation myocardial viability and cardiac function. The method is accurate, safe, and provides great spatial resolution as well as sensitivity.^[30]^ CMR has been used to assess myocardial viability throughout the previous decade. According to Cardona et al., CMR accurately detects myocardial viability in patients with reduced LVEF.^[17]^ We previously demonstrated that, using PET as gold standard, CMR has high consistency, sensitivity, and specificity for the detection of myocardial viability compared with PET in CTO patients.^[31]^ Therefore, in the SOS-moral trial, CMR will be used to evaluate both the amount of viable myocardial tissue and level of cardiac functioning.

## 4 Conclusions

SOS-moral will sort myocardial viability before randomization to avoid the effect of viability of myocardial on cardiac function. Hence, the real benefits of CTO-PCI on cardiac function among these patients without 100% survival viable myocardial will be assessed. Furthermore, the present trial strictly recruits patients with MVD and concurrent CTO. SOS-moral will provide evidence for the value of CTO-PCI among these selected patients. Nevertheless, there remain several potential limitations in this study. First, SAQ may be affected by factors other than recanalization of CTO artery during the study period. Second, patients with high risk such as those with left main artery disease stenosis ≥50% and severe arrhythmia are not included. Patients with left main artery disease are not eligible because the PCI procedure needs to be individualized. Because cardiac function might be affected by the severe arrhythmia, those with severe arrhythmia will be excluded. Therefore, the outcomes are unable to include all patients at high risk. Third, in a limited study period, the study may be not powered to detected MACE differences between the two groups. Therefore, further studies including high-risk patients and with longer follow-up periods are needed in the future.

## Acknowledgments

The authors wish to thank Ya Yang and Yueli Wang for their contribution in performing transthoracic echocardiography. The authors highly thank for Wenyi Zhang and Haoran Xing, who help recruit patients. We gratefully acknowledge Jiahui Li for performing follow-up evaluations of patients, as well as Xin Zhao and Nan Nan for collecting data. The authors also wish to thank the patients who participated in the study and staff from the 3 centers who supported the study.

## Author contributions

Xiantao Song and Rongchong Huang contributed to the study design. Jinfan Tian and Dongfeng Zhang contributed to draft the manuscript. Xueyao Yang contributes to the patients’ follow-up. Minduo Zhang and Yuan Zhou perform the intervention implementation and patients’ recruitment. Lijun Zhang contributes to CMR perform. Yi He and Hongzhi Mi help to CMR technology supervision. Huijuan Zuo contribute to the data monitor. Xiantao Song and Min Zhang contribute to the endopoint adjudication.

**Conceptualization:** Xiantao Song and Rongchong Huang.

**Data monitor:** Huijuan Zuo.

**Date management:** Jinfan Tian and Dongfeng Zhang.

**Endpoint adjudication:** Xiantao Song and Min Zhang.

**Investigation:** Xueyao Yang, Minduo Zhang and Yuan Zhou.

**Technology supervision:** Yi He and Hongzhi Mi.
